# Effect of anakinra on cytokine storm during sepsis-induced multiple organ dysfunction syndrome: case report and preclinical investigation

**DOI:** 10.3389/fimmu.2026.1904924

**Published:** 2026-08-19

**Authors:** Rebecca M. Blade, Sarah P. Lecher, Todd M. Umstead, Zissis C. Chroneos, Cainan Riccio-Baum, S. Rameeza Allie, Timothy J. Hahn, Daniel J. McKeone, Jessica E. Ericson, Theodore DeMartini, Debbie Spear, E. Scott Halstead

**Affiliations:** 1 Department of Pediatrics, Penn State College of Medicine, Hershey, PA, United States; 2 Division of Pediatric Critical Care Medicine, Penn State College of Medicine, Hershey, PA, United States; 3 Pulmonary Immunology and Physiology Laboratory, Penn State College of Medicine, Hershey, PA, United States; 4 Division of Neonatal-Perinatal Medicine, Penn State College of Medicine, Hershey, PA, United States; 5 Department of Cell and Biological Systems, Penn State College of Medicine, Hershey, PA, United States; 6 Division of Pediatric Rheumatology, Penn State College of Medicine, Hershey, PA, United States; 7 Dysregulated Immune Response Team (DIRT), Penn State College of Medicine, Hershey, PA, United States; 8 Division of Pediatric Hematology and Oncology, Penn State College of Medicine, Hershey, PA, United States; 9 Division of Pediatric Infectious Disease, Penn State College of Medicine, Hershey, PA, United States; 10 Department of Molecular and Precision Medicine, Penn State College of Medicine, Hershey, PA, United States

**Keywords:** anakinra, cytokine storm syndrome, HLH, MAS, sepsis, MODS

## Abstract

We describe the case of a young female who presented with apparent human metapneumovirus-associated illness and dehydration, but whose condition rapidly worsened with progression to multiple organ dysfunction syndrome (MODS). Multiple modalities were used to treat the patient, including: 1) extracorporeal life support (ECLS) for refractory shock; 2) anakinra, a human recombinant interleukin (IL)-1 receptor antagonist, for presumed cytokine storm; and 3) therapeutic plasma exchange (TPE) for thrombocytopenia-associated multiple organ failure (TAMOF). The patient’s organ dysfunction rapidly improved. *Post hoc* cytokine analysis revealed that anakinra administration was associated with marked decreases in interleukin (IL)-6 and IL-8 levels, but not in other cytokine biomarkers, indicating a possible upstream IL-1 effect on IL-6/IL-8 levels. Preclinical cytokine-based sepsis models were used to investigate this possibility. The preclinical results suggest that IL-6 levels are a function of synergistic IL-1 and tumor necrosis factor (TNF) activity. Further study is needed to determine appropriate patient populations, timing, and dosing of anakinra for suppressing IL-1 signaling during cytokine storm-induced MODS.

## Case report

The patient is a previously healthy 9-year-old female who presented to the emergency department with a viral illness characterized by fever and sore throat. A rapid group A streptococcal swab was negative, but the respiratory pathogen panel detected human metapneumovirus. Laboratory values demonstrated an elevated creatinine level (1.68 mg/dL), which was initially attributed to dehydration. The patient was admitted to the ED observation unit for intravenous fluid and rehydration therapy.

On hospital day (HD) 1, the patient was transferred to the PICU for increased work of breathing. On arrival at the PICU, the patient was noted to be in fulminant shock characterized by tachycardia, tachypnea, and poor perfusion with cyanotic distal extremities. Blood gas analysis demonstrated a mixed acidosis (pH 7.06, pCO_2_ 57.5 mmHg, base deficit −14). The patient was intubated, central venous access was obtained, and an arterial catheter was placed for continuous blood pressure monitoring, blood gas analysis, and laboratory sampling. The comprehensive metabolic panel demonstrated hyponatremia (Na 133 mEq/L), and the complete blood count (CBC) demonstrated thrombocytopenia (platelets 69 × 10^3^/µL) and leukopenia (white blood cells 2.09 × 10^3^/µL) without neutropenia (absolute neutrophil count 1.57 × 10^3^/µL). Inflammatory biomarkers demonstrated a slightly elevated ferritin level (301.7 ng/mL), elevated C-reactive protein (CRP; 11.7 mg/dL), and a significantly elevated procalcitonin (>100 ng/mL) level.

The patient was resuscitated with a total of 120 mL/kg of crystalloid and colloid bolus fluids over 6 h due to persistently low central venous pressures, presumably from profound capillary leak syndrome. The patient’s perfusion did not improve, and blood pressures continued to decrease despite the administration of inotrope infusions, including epinephrine (0.2 mcg/kg/min) and dobutamine (10 mcg/kg/min). Therefore, the patient was cannulated for venoarterial extracorporeal life support (ECLS) due to refractory shock. The calculated Pediatric Risk of Mortality III (PRISM III) (1) score for the patient was 36, which corresponded to a 67% risk of mortality.

The Penn State University Department of Pediatrics Dysregulated Immune Response Team (DIRT), a group of pediatric subspecialists with expertise in hyperinflammatory disease processes, was consulted. The patient’s fulminant shock fit a pattern of sepsis with cytokine storm. Thus, she was empirically administered the IL-1 receptor antagonist anakinra at a dose of 100 mg every 12 h (8 mg/kg/day) for a total of 8 days.

The patient also had signs of disseminated intravascular coagulation (DIC) with thrombocytopenia (platelets 20 × 10^3^/µL), an elevated international normalized ratio (INR) of 2.8, and a blood smear showing “few” schistocytes. ADAMTS13 activity was not available early in the hospital course (subsequent ADAMTS13 activity on HD 3 was 28%). The patient was diagnosed with thrombocytopenia-associated multiple organ failure (TAMOF) (2, 3), and therapeutic plasma exchange (TPE) was administered as an additional treatment modality for her MODS. The American Society for Apheresis (ASFA) guidelines for TPE in patients with sepsis-induced MODS are a Category III, Grade 2A indication with a recommendation of an individualized decision regarding the potential benefit of exchange (4). She received a 1.5× volume exchange with fresh frozen plasma (FFP) on HD 2, followed by 1× volume exchanges with FFP on HD 3 and 4. Her INR peaked at 2.8 on HD 1 and normalized to an INR of 1.0 by HD 4, while her platelet count nadir (20 × 10^3^/µL) was reached on HD 2 but recovered to >80 × 10^3^/µL by HD 4.

The patient’s hemodynamics improved, and she was decannulated from ECLS on HD 5. The perfusion to her digits improved, and no amputation was required. She remained on invasive mechanical ventilation until HD 9. She was downgraded from pediatric intensive level of care on HD 12 and discharged to inpatient rehabilitation on HD 20. Approximately 6 weeks after hospitalization, she was fully functional and playing sports.

**Figure 1:**
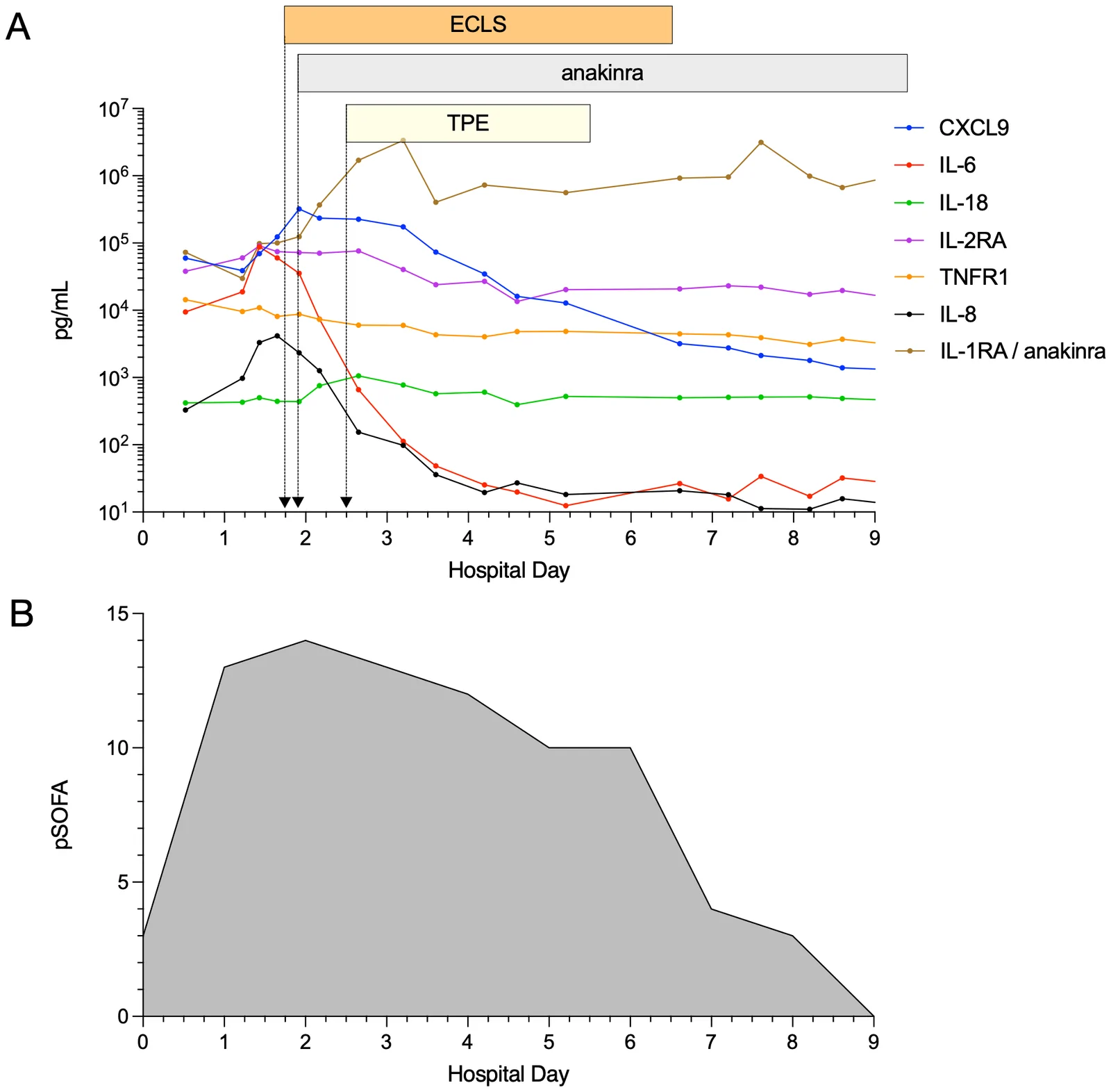
Cytokine levels and timing of interventions throughout sepsis-induced MODS. The patient presented to the pediatric intensive care unit (PICU) on hospital day 1 in refractory shock and was rapidly placed on extracorporeal life support (ECLS). The IL-1 receptor antagonist, anakinra, was empirically administered soon after ECLS. Therapeutic plasma exchange (TPE) was initiated on hospital day 2. Remnant citrated plasma clinical samples were collected from the clinical laboratory and cytokines were measured *post hoc* using the Ella™ automated immunoassay system (BioTechne) **(A)**. Pediatric sequential organ failure assessment (pSOFA) scores were calculated daily **(B)**.

The patient was enrolled in our ongoing, IRB-approved observational study, “Systemic Immune Profiles of Sepsis (SIPS; STUDY00005513). The protocol allows for the collection of remnant samples from the clinical laboratory for *post hoc* analysis. Remnant plasma (EDTA, lavender) samples were collected and stored at −80 °C before batch analysis. Selected sepsis biomarkers, including CXCL9, sIL-2RA, IL-6, IL-8, IL-18, and sTNFR1 were measured using custom cartridges on the Ella™ automated immunoassay system (Bio-Techne). Cytokine levels (note the exponential scale) throughout the hospitalization were plotted against the timing of interventions (**Figure 1A**). The cytokines IL-2RA and TNFR1 demonstrated gradual decreases over time. CXCL9, an interferon-induced chemokine, peaked just after ECLS initiation, and levels then fell gradually. In contrast, IL-8 and IL-6 exhibited dramatic decreases of 100- to 1,000-fold, respectively, from their peaks just prior to ECLS initiation and anakinra administration. We also measured plasma levels of IL-1 receptor antagonist (IL-1RA) using the Ella immunoassay system, with the foreknowledge that this would reveal the summation of endogenous and exogenous (anakinra) IL-1RA after anakinra administration (**Figure 1A**). The patient’s daily pediatric sequential organ failure assessment (pSOFA) (5) scores peaked on HD 2 and then fell to zero by HD 9 (**Figure 1B**).

## Preclinical investigation of pairwise cytokine sepsis models

We were surprised by the effect of anakinra on IL-6 levels. Recently, Takahama et al. demonstrated, using a preclinical murine model, that the pairwise intravenous administration of the recombinant cytokine TNF, in addition to either IL-1, IL-18, or IFN-γ, led to organism-wide sepsis-like responses (6). Importantly, in their models, TNF was essential for sepsis, whereas IL-6 was noncontributory (6). We recreated the Takahama pairwise cytokine preclinical models in an effort to dissect the patterns of biomarker expression versus cytokine signaling pathways activated.

All animal experiments were approved by the Pennsylvania State University College of Medicine Institutional Animal Care and Use Committee (IACUC protocol PRAMS201647230). Wild-type C57BL/6 mice were purchased from The Jackson Laboratory (Bar Harbor, ME). Adult (8- to 10-week-old) mice, with equal representation of males and females, were used for all experiments. All recombinant mouse cytokines (IFN-gamma, IL-1-beta, IL-18, TNF) were purchased from R&D Systems and lyophilized cytokines were resuspended in phosphate-buffered saline (PBS) on the day of injection. Mice were anesthetized with isoflurane, and cytokines were then administered intravenously via retro-orbital injection. Blood (approximately 10 μL) was collected in PBS with EDTA via serial tail sampling at predetermined time points (3 h, 6 h, 24 h, and 48 h), and plasma was stored at −80°C until batch analysis. Mice were weighed daily until recovery. The Ella™ automated microfluidic immunoassay system and mouse Simple Plex assays were used to measure mouse IL-1-beta, TNF, IL-6, and IFN-γ. All cytokines were measured at a final dilution of 1:100. CXCL9 was measured using DuoSet enzyme-linked immunosorbent assay (ELISA) kits (R&D Systems), and cytokine concentrations were quantified using a microplate reader set at a wavelength of 450 nm (SpectraMax M5, Molecular Devices).

**Figure 2:**
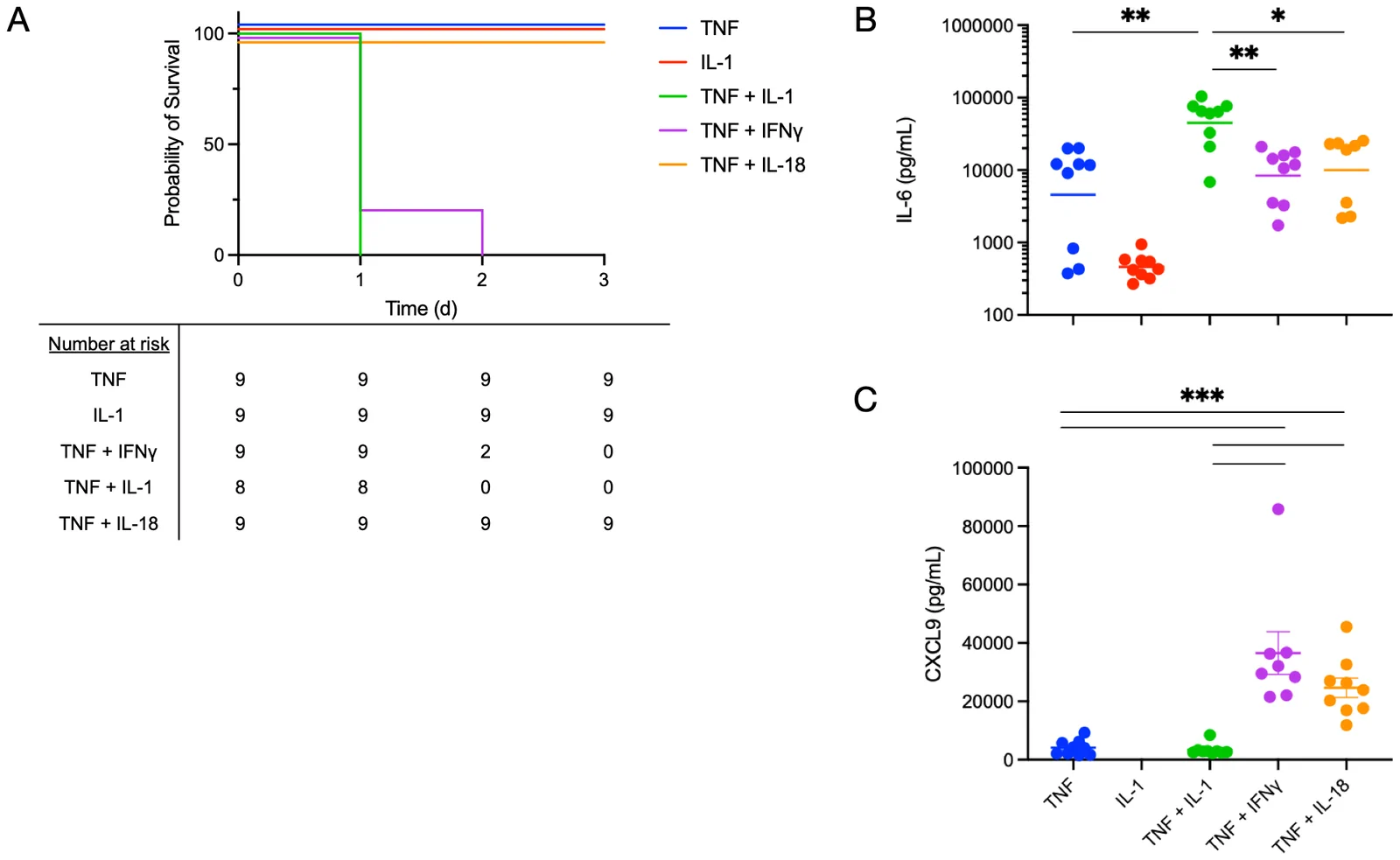
Preclinical modeling of pairwise cytokine-mediated sepsis. Survival curves generated after the I.V. administration of cytokines to young adult C57BL6/J mice via retro-orbital injection at time 0 **(A)**. Results of three independent experiments with n = 3 mice per group per experiment using relatively equal numbers of female and male mice. The administered dose was 2.5 μg per cytokine. The combinations of TNF + IL-1 beta and TNF + IFNγ led to rapid death **(A)**. The resulting IL-6 **(B)** and CXCL9 **(C)** levels at 6 h (0.25 days) after cytokine administration. *p < 0.05, **p < 0.01,***p < 0.001.

In agreement with Takahama et al., the administration of TNF + IL-1 or TNF + IFNγ was universally fatal (**Figure 2A**, p <0.0001), while the administration of TNF and TNF + IL-18 led to weight loss (**Supplementary Figure 1**) but not mortality. We investigated the biomarker profiles of the cytokine pairwise combinations by measuring plasma cytokine levels at multiple time points after administration (3 h, 6 h, 24 h, and 48 h) by serial tail vein sampling. However, due to the rapid mortality conveyed by the pairings of TNF + IL-1 and TNF + IFNγ, only samples from early time points were available for all mice. Thus, we focused on the early 3 h and 6 h time points. Unsurprisingly, the administered cytokines (TNF, IL-1, IFNγ) were elevated in their respective groups (**Supplementary Figures 2–4**). IL-6 levels were increased by the administration of TNF across all groups, but, interestingly, IL-6 levels were significantly higher in the group also receiving IL-1 (**Figure 2B**). Given the role of CXCL9 as a marker of IFNγ signaling (7) and the ability of IL-18 to drive IFNγ production (8), levels of CXCL9 were elevated by the administration of both IFNγ (9) and IL-18 (10) (**Figure 2C**).

**Figure 3:**
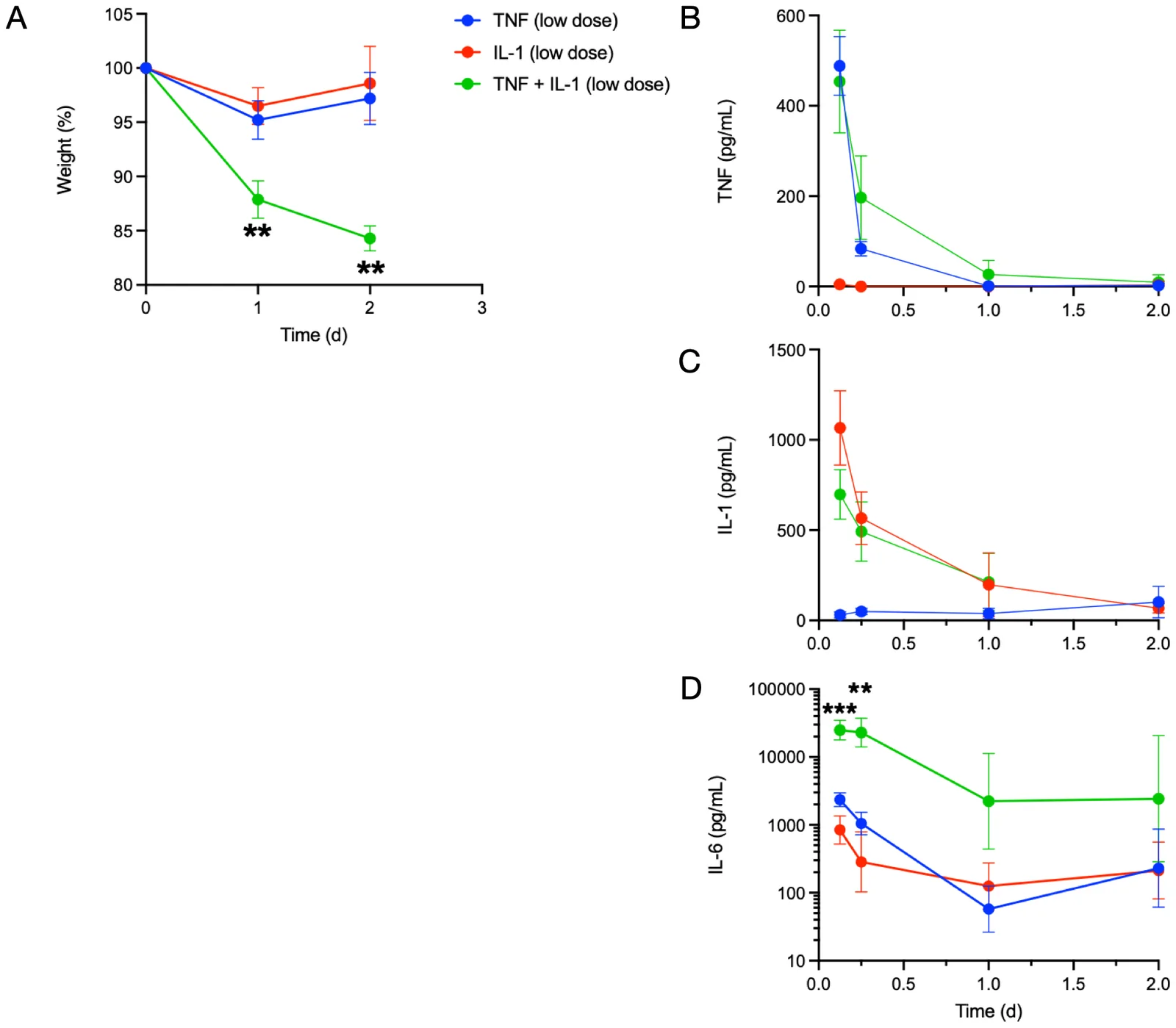
Investigation of relationship between IL-1, TNF, and IL-6 expression using preclinical pairwise model. Lower dosages (1 μg) of TNF and IL-1 were administered and the combination of TNF + IL-1 led to significantly more weight loss than either TNF or IL-1 alone **(A)**. Serial tail vein bleeding was used establish a time course of cytokines levels. TNF **(B)** and IL-1 **(C)** levels fell rapidly following administration. IL-6 levels were significantly higher when both TNF and IL-1 were administered a **(D)** as compared to TNF and IL-1 alone. *p < 0.05, **p < 0.01,***p < 0.001.

We leveraged the pairwise cytokine preclinical model to elucidate the relationship between IL-1, TNF, and IL-6. We administered sublethal cytokine dosages (1 μg) of each cytokine (TNF, IL-1, or both) and measured subsequent cytokine levels by serial tail vein sampling. While mice that received the low-dose combination TNF + IL-1 lost significant weight (**Figure 3A**), no low dose cytokine-treated mice died. Not surprisingly, TNF and IL-1 levels corresponded with their administration and demonstrated simple pharmacokinetics (**Figures 3B, C**). Mice administered both TNF + IL-1 demonstrated significantly higher IL-6 levels than those administered either TNF or IL-1 alone (**Figure 3D**).

## Discussion

We used multiple modalities to support this patient, including ECLS, immune modulation with anakinra, and therapeutic plasma exchange. We cannot and do not propose that any one treatment was the reason for her rapid and complete recovery. The patient’s hemodynamics and maintenance of organ perfusion during septic shock were supported by ECLS. The use of ECLS is becoming more common as a last resort in pediatric sepsis-induced shock (11). Second, TPE was considered for the management of TAMOF (2, 3) or sepsis/MAS-induced thrombotic microangiopathy (12, 13). Lastly, we chose to initiate IL-1 blockade very early in the clinical course of sepsis-induced MODS. Interestingly, sepsis was the chief indication in the original studies of IL-1 receptor blockade. IL-1 receptor antagonism has been described as beneficial in both murine (14) and primate (15) preclinical models of sepsis. Furthermore, a dose-related survival benefit was observed with IL-1 antagonism, specifically in patients with septic shock (as opposed to sepsis without shock), gram-negative infection, and elevated IL-6 concentrations (16). The ongoing NICHD-supported Targeted Reversal of Inflammation in Pediatric Sepsis-induced MODS (TRIPS) trial (17) is examining the role of IL-1 blockade using anakinra to decrease organ dysfunction (NCT05267821). The TRIPS study administers anakinra starting on MODS day 2. The present case report demonstrates a positive outcome with very early administration of anakinra. Future studies should address the effects of the timing of anakinra administration on sepsis outcomes.

This case highlights several interesting points pertaining to the diagnosis and treatment of cytokine storm/shock. Interestingly, while the patient demonstrated the clinical appearance of bacterial septic shock, all cultures were negative other than a tracheal aspirate obtained hours after intubation that grew methicillin-sensitive *Staphylococcus aureus* (MSSA). The tracheal aspirate Gram stain demonstrated 4+ gram-positive cocci in clusters, a few gram-negative rods, and a few neutrophils. However, the patient never fit a diagnosis of pneumonia, as she did not demonstrate infiltrates on chest radiograph, nor was poor oxygenation noted. It is possible that the patient had translocation of immunostimulatory bacterial products, such as lipopolysaccharide (LPS) or lipoteichoic acid (LTA), across mucous membranes. Preclinical models demonstrate that viral infection predisposes to exaggerated responses to bacterial products and can mimic secondary hemophagocytic lymphohistiocytosis (HLH) (18). To investigate the possibility that this patient met criteria for HLH and/or MAS, we compared the HLH-2004 (19, 20), H-Score (21), and Ravelli criteria for sJIA/MAS (22). While the patient did meet criteria for MAS, she did not meet criteria for HLH (**Table 1**).

**Table 1:** Hemophagocytic lymphohistiocytosis (HLH) and macrophage activation syndrome (MAS) scoring.

Criteria	Patient values	HLH-2004		H-score		MAS (Ravelli criteria)	
		Criteria	Score	Criteria	Score	Criteria	Score
Fever	40.5 °C	>38.5 °C	+		49		
Splenomegaly/Organomegaly	None		–		0		
Cytopenias (>1 lineage)			–		0		
- Hemoglobin	9.1	<9 g/dL					
- Platelets	19	<100 K/μL				<181 K/μL	+
- Neutrophils	1.35	<1 K/μL					
Hypertriglyceridemia and/or Hypofibrinogenemia			–				
- Hypertriglyceridemia	Not obtained	>265 mg/dL			0	>156 mg/dL	–
- Hypofibrinogenemia	229	<150 mg/dL			30	<360 mg/dL	+
Hemophagocytosis	Not obtained	None	–				
Low/absent NK cell activity	Not obtained		–				
Ferritin	1558	>500 ng/mL	+		0	>684 ng/mL	+
sCD25	Not obtained	>2,400 U/mL	–				
AST	72			<30 U/L	19	>48 U/L	+
Genetics	Not obtained						
Total			2 of 8		98		
		Not HLH		~1% probability HLH		Meets criteria for MAS	

In terms of routine clinical inflammatory laboratory values, her C-reactive protein (CRP) (11.73 mg/dL) and procalcitonin (>100 ng/mL) levels peaked on HD 1, whereas her ferritin (1,558 ng/mL) level peaked on HD 2. While some studies are using CRP levels alone to cohort “inflamed” patients (17), another study (NCT05914454) is investigating the role of anakinra in adult acute respiratory distress syndrome (ARDS) using *either* elevated IL-6 (>80 pg/mL) or CRP (>25 mg/dL) as inclusion criteria. This approach, using either IL-6 or CRP levels for inclusion, is probably wise. CRP levels, while readily measurable, may not be reliable in the setting of immune-related liver injury (23). CRP is produced by hepatocytes, but impaired liver synthetic function may result in artificially low CRP levels despite high IL-6 signaling (24). Our patient demonstrated this discrepancy between IL-6 and CRP levels: on HD 1 the patient’s IL-6 level was 18,731 pg/mL whereas her CRP was only 11.73 mg/dL. Subsequent testing showed an even higher IL-6 level (82,431 pg/mL), whereas the CRP had actually fallen (8.26 mg/dL) at the same time point. That being said, the primary factor underlying discrepancies in the correlations among IL-6, procalcitonin, and CRP is differences in their blood concentration pharmacokinetics (25). Further study is needed regarding the role of IL-6 levels in directing the early initiation of anakinra, and other modalities, for sepsis-induced organ failure.

Our clinical data support the notion that anakinra blockade of IL-1 specifically affected IL-6 and IL-8 levels. IL-8 is actually a chemokine, C-X-C motif chemokine ligand 8 (CXCL8). IL-6 and IL-8 levels are known to be tightly correlated (26); however, mice do not express IL-8. In our simple murine cytokine-induced sepsis model, IL-6 expression appears to be downstream of synergistic IL-1 and TNF signaling activity. In 1992, Damas et al. demonstrated that IL-6 appears to be a good marker of disease severity during bacterial infection, as opposed to IL-1β (27). These data are supported by a preclinical study showing IL-6 to be a biomarker of IL-1 signaling activity (28). The strength of the relationship between IL-1 signaling and IL-6 levels was previously described in the initial primate preclinical trials of IL-1 blockade (15). This association was further strengthened by the initial clinical trial of human recombinant IL-1 receptor alpha chain (16), which showed a dose-dependent survival benefit in patients with septic shock, with Gram-negative infection, and in patients with elevated IL-6 levels at study entry. Furthermore, the magnitude of the decrease in IL-6 concentration 24 h after the initiation of therapy was correlated with an increasing IL-1ra treatment dose (15). Lastly, two studies of anakinra administration in the setting of COVID-19 demonstrated that IL-6 levels decreased in the group treated with anakinra (29) and that the initial IL-6 level was predictive of survival (30).

This case report and preclinical study have several limitations. Because the patient received multiple treatment modalities in quick succession, it is not possible to dissect out the relative contributions to the positive outcome. What is compelling about this case is the demonstration of the effects of the interventions on the patient’s cytokine profile over time. During the MODS course, IL-6 and IL-8 levels decreased more quickly than the other cytokines/biomarkers. While this could be a byproduct of cytokine clearance by TPE, we believe this to be a specific effect of anakinra, as nonspecific cytokine clearance by TPE would have additionally caused decreases in CXCL9, IL-2RA, IL-18, and TNFR1 levels. Additionally, IL-1RA levels increased approximately 10-fold following the initiation of anakinra. These results make sense. as TPE only performs 1–1.5× volume exchanges at a time and would theoretically only decrease cytokine levels by 50%–75% and was administered once daily. Other modalities that may be equally beneficial in sepsis-induced cytokine storm include cytokine-adsorbing hemofiltration (31–33) for nonspecific cytokine depletion and direct hemoperfusion using a polymyxin B-immobilized fiber column (PMX-DHP) for the absorption of endotoxin (34). Future precision medicine-based studies are needed for predictive enrichment of these various modalities.

In total, this case and these preclinical results suggest that aggressive critical care with multiple treatment modalities can prove beneficial and that IL-6 may prove efficacious as a biomarker that represents ongoing IL-1 and TNF signaling activity. Future studies examining the optimal dosing of anakinra and the predictive enrichment of patients using IL-6 are needed. Indeed, as hyperinflammatory ARDS (35) and sepsis (36) are traditionally defined, they may partly reflect dysregulated IL-1 signaling; anakinra is an attractive potential therapy to test in this subphenotype (37, 38).

## Data Availability

The original contributions presented in the study are included in the article/**Supplementary Material**. Further inquiries can be directed to the corresponding author.

## References

[1] Pollack MM Patel KM Ruttimann UE . PRISM III: an updated pediatric risk of mortality score. Crit Care Med. (1996) 24:743–52. doi: 10.1097/pcc.0000000000000558 8706448

[2] Nguyen TC Han YY Kiss JE Hall MW Hassett AC Jaffe R . Intensive plasma exchange increases a disintegrin and metalloprotease with thrombospondin motifs-13 activity and reverses organ dysfunction in children with thrombocytopenia-associated multiple organ failure. Crit Care Med. (2008) 36:2878–87. doi: 10.1016/j.ccc.2019.12.010 18828196 PMC2772176

[3] Fortenberry JD Nguyen T Grunwell JR Aneja RK Wheeler D Hall M . Therapeutic plasma exchange in children with thrombocytopenia-associated multiple organ failure: the thrombocytopenia-associated multiple organ failure network prospective experience. Crit Care Med. (2019) 47:e173–81. doi: 10.1097/ccm.0000000000003559 30531184 PMC9084562

[4] Connelly-Smith L Alquist CR Aqui NA Hofmann JC Klingel R Onwuemene OA . Guidelines on the use of therapeutic apheresis in clinical practice - evidence-based approach from the writing committee of the American Society for Apheresis: the ninth special issue. J Clin Apher. (2023) 38:77–278. doi: 10.1002/jca.22043 37017433

[5] Matics TJ Sanchez-Pinto LN . Adaptation and validation of a pediatric sequential organ failure assessment score and evaluation of the sepsis-3 definitions in critically ill children. JAMA Pediatr. (2017) 171:e172352. doi: 10.1001/jamapediatrics.2017.2352 28783810 PMC6583375

[6] Takahama M Patil A Richey G Cipurko D Johnson K Carbonetto P . A pairwise cytokine code explains the organism-wide response to sepsis. Nat Immunol. (2024) 25:226–39. doi: 10.1038/s41590-023-01722-8 38191855 PMC10834370

[7] Rocco JM Oved JH Patel RJ Herskovits AZ Nair N Shakoory B . CXCL9 as a novel prognostic marker to identify high-risk adults with hemophagocytic lymphohistiocytosis. Blood. (2026) 147:960–72. doi: 10.1182/blood.2025030976 41369611 PMC13000932

[8] Fantuzzi G Puren AJ Harding MW Livingston DJ Dinarello CA . Interleukin-18 regulation of interferon gamma production and cell proliferation as shown in interleukin-1beta-converting enzyme (caspase-1)-deficient mice. Blood. (1998) 91:2118–25. doi: 10.1182/blood.v91.6.2118.2118_2118_2125 9490698

[9] Sgadari C Farber JM Angiolillo AL Liao F Teruya-Feldstein J Burd PR . Mig, the monokine induced by interferon-gamma, promotes tumor necrosis *in vivo*. Blood. (1997) 89:2635–43. doi: 10.1182/blood.v89.8.2635 9108380

[10] Kanda N Shimizu T Tada Y Watanabe S . IL-18 enhances IFN-gamma-induced production of CXCL9, CXCL10, and CXCL11 in human keratinocytes. Eur J Immunol. (2007) 37:338–50. doi: 10.1002/eji.200636420 17274000

[11] Melnikov G Grabowski S Broman LM . Extracorporeal membrane oxygenation for septic shock in children. ASAIO J. (2022) 68:262–7. doi: 10.1097/mat.0000000000001464 34156196 PMC8797002

[12] Vincent JL Castro P Hunt BJ Jorres A Praga M Rojas-Suarez J . Thrombocytopenia in the ICU: disseminated intravascular coagulation and thrombotic microangiopathies-what intensivists need to know. Crit Care. (2018) 22:158. doi: 10.1097/00000542-196601000-00029 29895296 PMC5998546

[13] Minoia F Tibaldi J Muratore V Gallizzi R Bracaglia C Arduini A . Thrombotic microangiopathy associated with macrophage activation syndrome: a multinational study of 23 patients. J Pediatr. (2021) 235:196–202. doi: 10.1016/j.jpeds.2021.04.004 33836183

[14] Alexander HR Doherty GM Buresh CM Venzon DJ Norton JA . A recombinant human receptor antagonist to interleukin 1 improves survival after lethal endotoxemia in mice. J Exp Med. (1991) 173:1029–32. doi: 10.1084/jem.173.4.1029 1826127 PMC2190820

[15] Fischer E Marano MA Van Zee KJ Rock CS Hawes AS Thompson WA . Interleukin-1 receptor blockade improves survival and hemodynamic performance in Escherichia coli septic shock, but fails to alter host responses to sublethal endotoxemia. J Clin Invest. (1992) 89:1551–7. doi: 10.1172/jci115748 1533231 PMC443028

[16] Fisher CJ Jr. Slotman GJ Opal SM Pribble JP Bone RC Emmanuel G . Initial evaluation of human recombinant interleukin-1 receptor antagonist in the treatment of sepsis syndrome: a randomized, open-label, placebo-controlled multicenter trial. Crit Care Med. (1994) 22:12–21. doi: 10.1097/00003246-199401000-00008 8124953

[17] VanBuren JM Hall M Zuppa AF Mourani PM Carcillo J Dean JM . The design of nested adaptive clinical trials of multiple organ dysfunction syndrome children in a single study. Pediatr Crit Care Med. (2023) 24:e635–46. doi: 10.1097/pcc.0000000000003332 37498156 PMC10817996

[18] Wang A Pope SD Weinstein JS Yu S Zhang C Booth CJ . Specific sequences of infectious challenge lead to secondary hemophagocytic lymphohistiocytosis-like disease in mice. Proc Natl Acad Sci USA. (2019) 116:2200–9. doi: 10.1073/pnas.1820704116 30674681 PMC6369774

[19] Henter JI Horne A Arico M Egeler RM Filipovich AH Imashuku S . HLH-2004: diagnostic and therapeutic guidelines for hemophagocytic lymphohistiocytosis. Pediatr Blood Cancer. (2007) 48:124–31. doi: 10.1056/nejmra2314005 16937360

[20] Henter JI Sieni E Eriksson J Bergsten E Hed Myrberg I Canna SW . Diagnostic guidelines for familial hemophagocytic lymphohistiocytosis revisited. Blood. (2024) 144:2308–18. doi: 10.1182/blood.2024025077 39046779 PMC11619794

[21] Fardet L Galicier L Lambotte O Marzac C Aumont C Chahwan D . Development and validation of the HScore, a score for the diagnosis of reactive hemophagocytic syndrome. Arthritis Rheumatol. (2014) 66:2613–20. doi: 10.1002/art.38690 24782338

[22] Ravelli A Minoia F Davi S Horne A Bovis F Pistorio A . 2016 classification criteria for macrophage activation syndrome complicating systemic juvenile idiopathic arthritis: a European League Against Rheumatism/American College of Rheumatology/Paediatric Rheumatology International Trials Organisation collaborative initiative. Arthritis Rheumatol. (2016) 68:566–76. doi: 10.1002/art.39332 26314788

[23] Ross Y Ballou S . Reliability of C-reactive protein as an inflammatory marker in patients with immune-mediated inflammatory diseases and liver dysfunction. Rheumatol Adv Pract. (2023) 7:rkad045. doi: 10.1093/rap/rkad045 37228508 PMC10203543

[24] Sproston NR Ashworth JJ . Role of C-reactive protein at sites of inflammation and infection. Front Immunol. (2018) 9:754. doi: 10.3389/fimmu.2018.00754 29706967 PMC5908901

[25] Meisner M . Pathobiochemistry and clinical use of procalcitonin. Clin Chim Acta. (2002) 323:17–29. doi: 10.1016/s0009-8981(02)00101-8 12135804

[26] Kesmez Can F Ozkurt Z Ozturk N Sezen S . Effect of IL-6, IL-8/CXCL8, IP-10/CXCL 10 levels on the severity in COVID 19 infection. Int J Clin Pract. (2021) 75:e14970. doi: 10.1111/ijcp.14970 34626520 PMC8646602

[27] Damas P Ledoux D Nys M Vrindts Y De Groote D Franchimont P . Cytokine serum level during severe sepsis in human IL-6 as a marker of severity. Ann Surg. (1992) 215:356–62. doi: 10.1097/00000658-199204000-00009 PMC1242452 1558416

[28] McGeough MD Pena CA Mueller JL Pociask DA Broderick L Hoffman HM . Cutting edge: IL-6 is a marker of inflammation with no direct role in inflammasome-mediated mouse models. J Immunol. (2012) 189:2707–11. doi: 10.4049/jimmunol.1101737 22904305 PMC3495128

[29] Kyriazopoulou E Poulakou G Milionis H Metallidis S Adamis G Tsiakos K . Early treatment of COVID-19 with anakinra guided by soluble urokinase plasminogen receptor plasma levels: a double-blind, randomized controlled phase 3 trial. Nat Med. (2021) 27:1752–60. doi: 10.1038/s41591-021-01499-z 34480127 PMC8516650

[30] Jackson LE Khullar N Beukelman T Chapleau C Kamath A Cron RQ . Prediction of survival by IL-6 in a randomized placebo-controlled trial of anakinra in COVID-19 cytokine storm. Viruses. (2023) 15(10):2036. doi: 10.3390/v15102036 37896812 PMC10612044

[31] Saeed A Jafarian H Amanati A . Effective management of pediatric septic shock: a case study utilizing continuous renal replacement therapy with cytosorb and citrate in a leukemic patient with hyper-interleukin (IL)-6-naemia and severe thrombocytopenia. BMC Infect Dis. (2025) 25:410. doi: 10.1186/s12879-025-10807-8 40140759 PMC11938599

[32] Hirasawa H Oda S Nakamura M Watanabe E Shiga H Matsuda K . Continuous hemodiafiltration with a cytokine-adsorbing hemofilter for sepsis. Blood Purif. (2012) 34:164–70. doi: 10.1159/000342379 23095416

[33] Bottari G Ranieri VM Ince C Pesenti A Aucella F Scandroglio AM . Use of extracorporeal blood purification therapies in sepsis: the current paradigm, available evidence, and future perspectives. Crit Care. (2024) 28:432. doi: 10.1186/s13054-024-05220-7 39722012 PMC11670469

[34] Neyra JA Legrand M Tidswell MA Al-Khafaji A Galphin C Rains R . Polymyxin B haemoadsorption in endotoxic septic shock (Tigris): a multicentre, open-label, Bayesian, randomised, controlled, phase 3 trial. Lancet Respir Med. (2026) 14:443–52. doi: 10.1016/s2213-2600(26)00047-0 41887242

[35] Sinha P Delucchi KL McAuley DF O'Kane CM Matthay MA Calfee CS . Development and validation of parsimonious algorithms to classify acute respiratory distress syndrome phenotypes: a secondary analysis of randomised controlled trials. Lancet Respir Med. (2020) 8:247–57. doi: 10.1016/s2213-2600(19)30369-8 31948926 PMC7543720

[36] Sinha P Kerchberger VE Willmore A Chambers J Zhuo H Abbott J . Identifying molecular phenotypes in sepsis: an analysis of two prospective observational cohorts and secondary analysis of two randomised controlled trials. Lancet Respir Med. (2023) 11(11):965–74. doi: 10.1016/s2213-2600(23)00237-0 37633303 PMC10841178

[37] Shakoory B Carcillo JA Chatham WW Amdur RL Zhao H Dinarello CA . Interleukin-1 receptor blockade is associated with reduced mortality in sepsis patients with features of macrophage activation syndrome: reanalysis of a prior phase III trial. Crit Care Med. (2016) 44:275–81. doi: 10.1097/ccm.0000000000001402 26584195 PMC5378312

[38] Meyer NJ Reilly JP Anderson BJ Palakshappa JA Jones TK Dunn TG . Mortality benefit of recombinant human interleukin-1 receptor antagonist for sepsis varies by initial interleukin-1 receptor antagonist plasma concentration. Crit Care Med. (2018) 46:21–8. doi: 10.1097/ccm.0000000000002749 28991823 PMC5734955

