# Supplementary material for: Effect of anakinra on cytokine storm during sepsis-induced multiple organ dysfunction syndrome: case report and preclinical investigation
Source: Front Immunol. 2026 Aug 19;17:1904924. doi: 10.3389/fimmu.2026.1904924 (PMC13533806; doi:10.3389/fimmu.2026.1904924)

Supplemental Figure 1

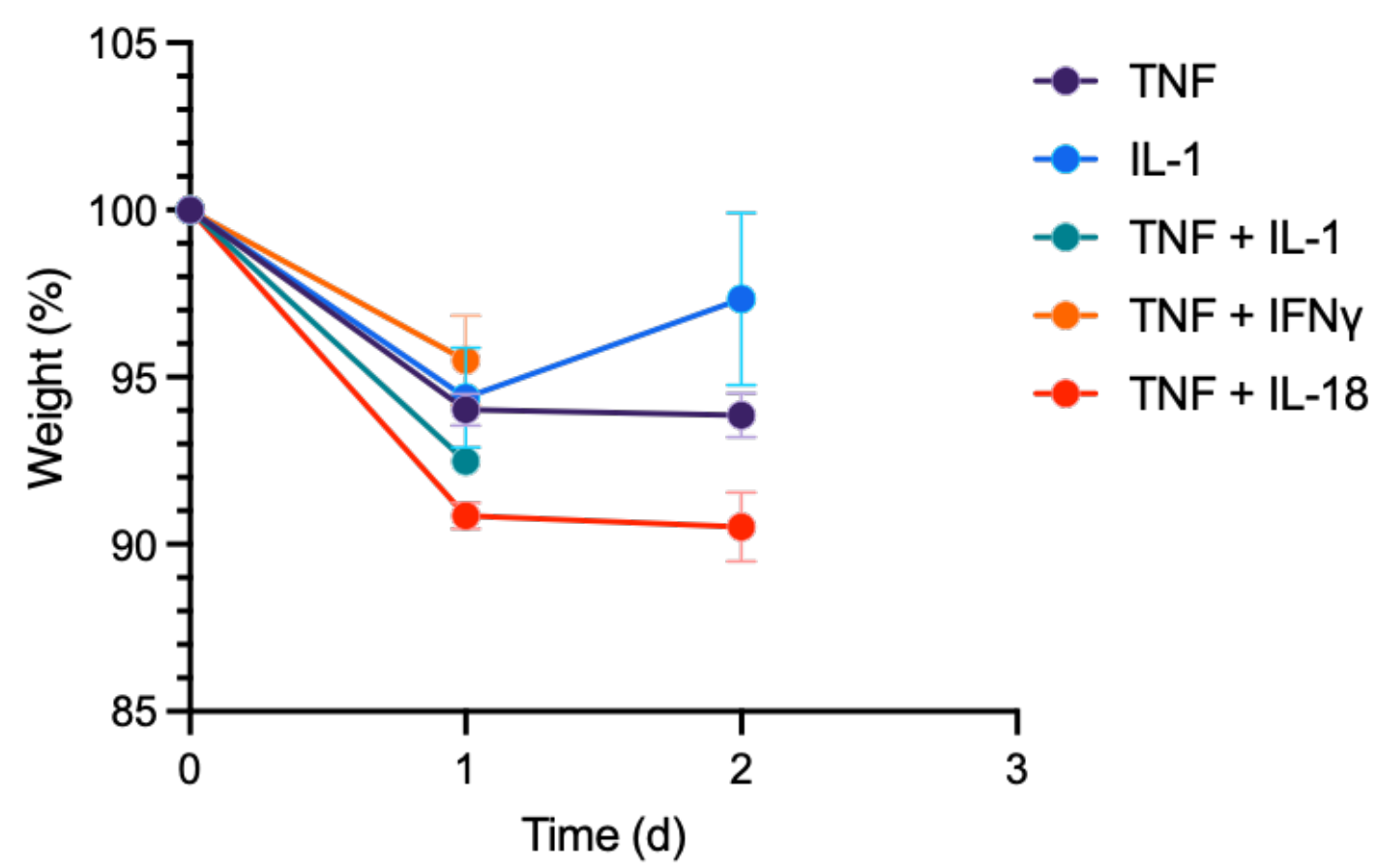

Supplemental Figure 2

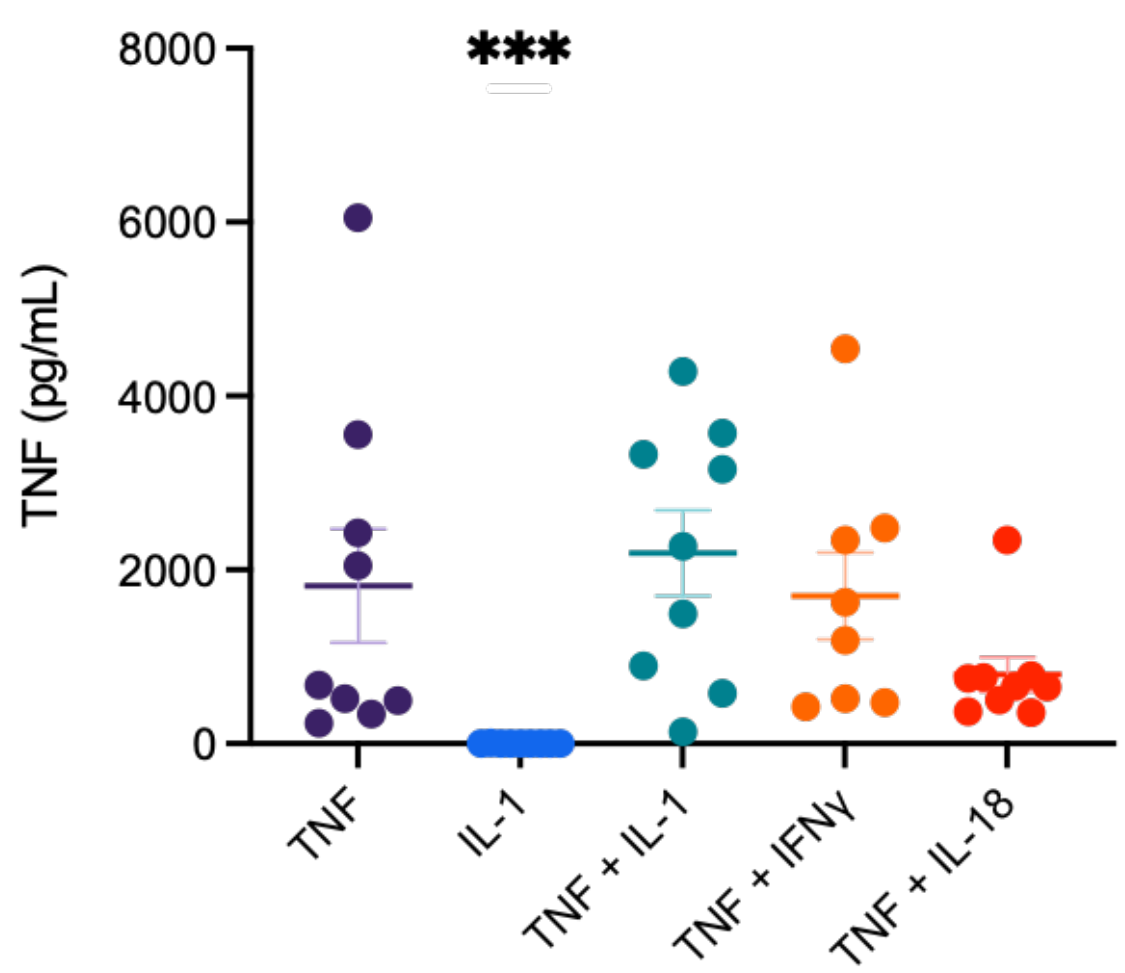

Supplemental Figure 3

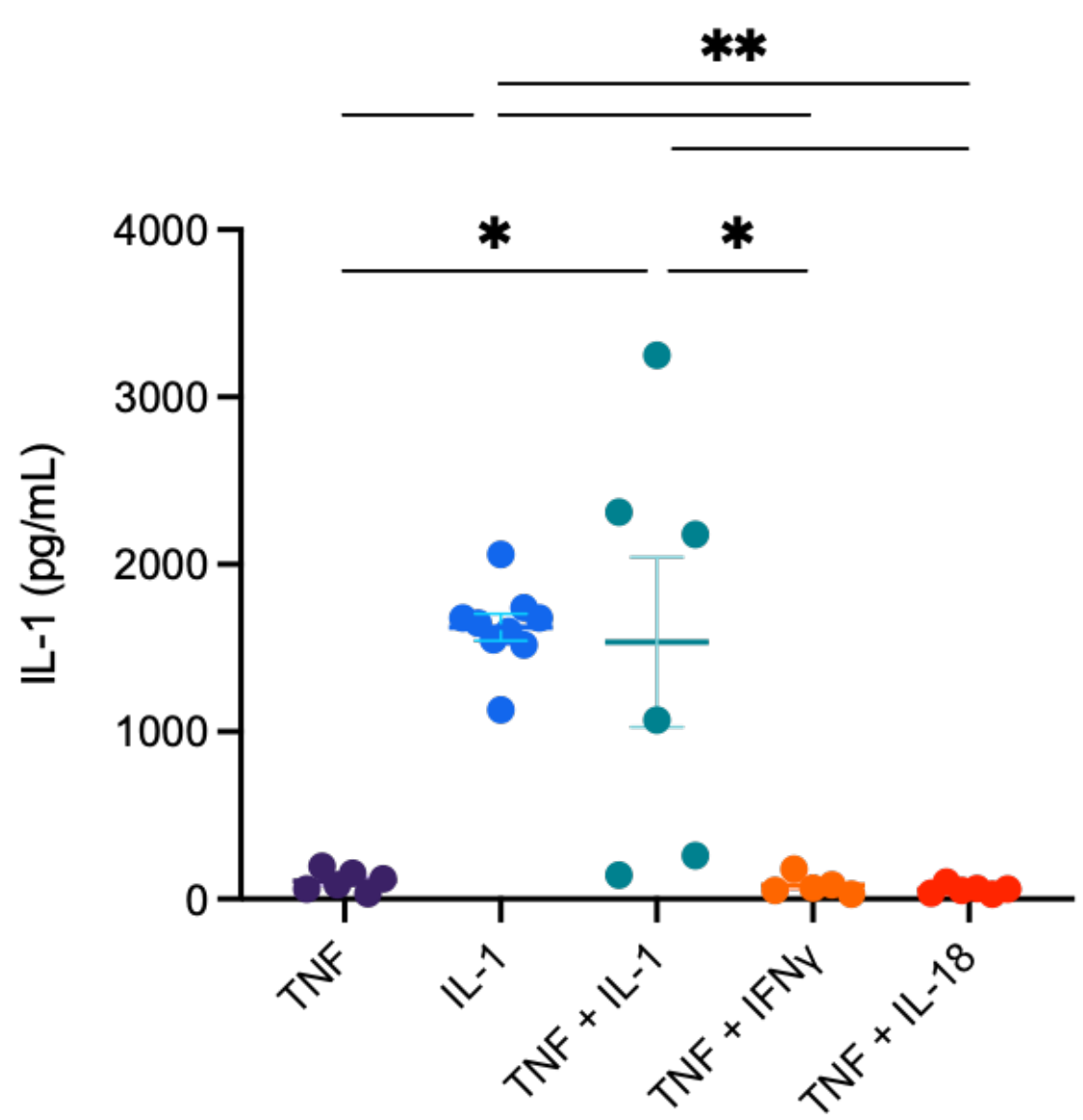

Supplemental Figure 4

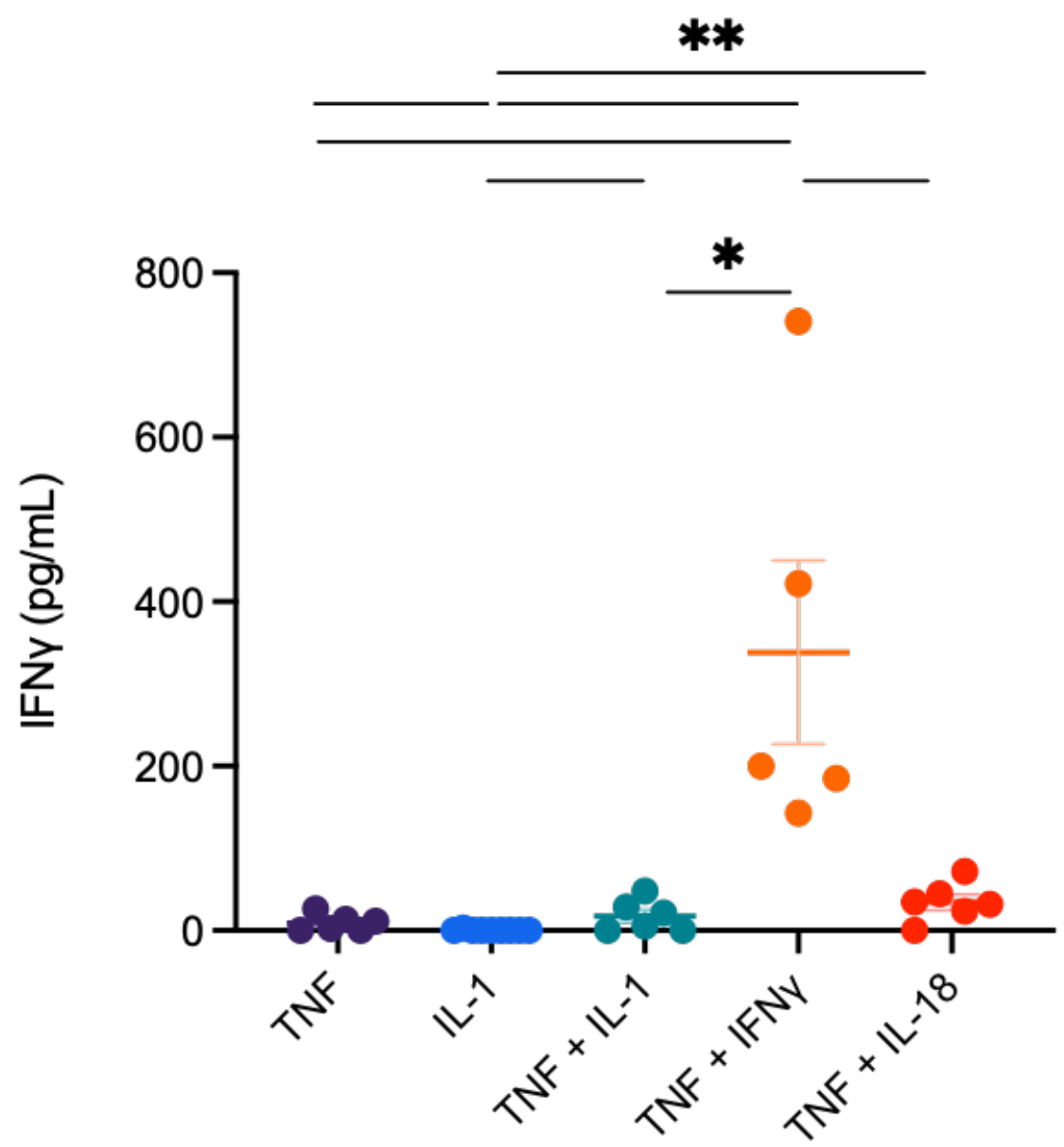

Supplement: Supplementary file 1 [file SupplementaryFile1.pdf]
